# Reviewing advancement in *Mycoplasma pneumoniae* P30 adhesin protein provides insights for future diagnosis and treatment

**DOI:** 10.3389/fmicb.2024.1515291

**Published:** 2024-12-13

**Authors:** Yingying Zuo, Ru Zhang, Shuihong Li

**Affiliations:** ^1^Hengyang Medical School, University of South China, Hengyang, China; ^2^The Seventh Affiliated Hospital of University of South China, Changsha, China

**Keywords:** *Mycoplasma pneumoniae*, P30 protein, adhesion, diagnosis, vaccine

## Abstract

*Mycoplasma pneumoniae* is a major pathogen that causes upper and lower respiratory tract infections in children, adolescents, and elderly individuals and can lead to pneumonia, intrapulmonary and extrapulmonary complications, and respiratory sequelae. *M. pneumoniae* must adhere to respiratory epithelial cells of a host for infection. The P1 and P30 proteins, as two adhesin proteins of *M. pneumoniae*, have attracted extensive attention from many researchers. In this paper, we present the latest research progress on the P30 protein in terms of structure and mutation typing, physiological function, clinical serological diagnosis and vaccine development in a literature review. This study deepens our knowledge on the pathogenesis of *M. pneumoniae* and is useful for diagnosing and preventing *M. pneumoniae* infection.

## 1 Introduction

Atypical pathogens, such as *Mycoplasma pneumoniae*, are major pathogens responsible for community-acquired pneumonia. *M. pneumoniae* is a naturally cell wall-less bacterium and can be transmitted by coughing or sneezing, causing inflammation of the upper and lower respiratory tract (e.g., pneumonia, bronchitis, tonsillitis, sinusitis, etc.) as well as extrapulmonary complications (e.g., mucocutaneous rash, nephritis, myocarditis, and meningoencephalitis.) in humans (Yu and Fei, 2016; Meyer Sauteur et al., 2020). Many patients may develop life-threatening severe lung injury, i.e., intrapulmonary complications (e.g., necrotizing pneumonia, pleural effusion, pulmonary embolism) (Sauteur et al., 2012). In recent years, with the increasing rate of drug resistance, the sequelae of refractory pneumonia caused by infection with drug-resistant strains of *M. pneumoniae* (e.g., chronic interstitial fibrosis, pulmonary atelectasis, bronchiectasis of the lungs, and occlusive fine bronchitis) can lead to a severe decline in lung function (Gao et al., 2019; Jiang et al., 2022; Suzuki et al., 2017).

The current knowledge on the pathogenic mechanism of *M. pneumoniae* is unclear, and researchers generally believe that its main pathogenic mechanisms include cell adhesion, immune escape, inflammatory response-induced immune damage, and cytotoxicity (Jiang et al., 2022; Jiang et al., 2021; Waites and Atkinson, 2009; Atkinson et al., 2008; Marshall et al., 1995). Among them, cell adhesion is a multifactorial and complex process, and *M. pneumoniae* mediates cell adhesion through a tip organelle at one end, in which the tip structure accumulates many adhesion proteins. Adhesion proteins of *M. pneumoniae* mainly include the P1, P30, P116, and P65 proteins, as well as auxiliary proteins P40 and P90 proteins (i.e., proteins B and C), and high molecular weight proteins HMW1-3 (Baseman et al., 1982; Balish, 2006; Widjaja et al., 2015; Hu et al., 1982; Vizarraga et al., 2020; Nakane et al., 2011; Franzoso et al., 1993; Seto et al., 2001; Seto and Miyata, 2003). The manifestation of these primary adhesion proteins in heterologous expression systems provides an opportunity to study the role of these proteins in pathogenicity and contributes to the development of diagnostic reagents, whereas auxiliary adhesion proteins mainly regulate the direction of movement and the distribution of primary adhesion proteins (Chaudhry, 2007). Dallo S F et al. first identified the P30 protein with a molecular weight of 30 kDa on the surface of *M. pneumoniae*; after the P1 protein, P30 was the second protein identified that is associated with the cell adhesion and virulence effects of *M. pneumoniae*. Among the major adhesion proteins, P1 has been the most extensively studied. 169 kDa of the P1 protein is involved not only in the adhesion process through receptor recognition but also in the gliding movement of *M. pneumoniae*, and the P1 protein is highly immunogenic, which is of great significance for serological diagnosis and vaccine development. Although relatively understudied, the P30 protein has been found to exhibit more structural similarity to the P1 protein than that of other major adhesion proteins; as a result, these two proteins perform similar functions. As a key adhesion protein, the P30 protein containing 275 amino acids not only plays an important role in cell adhesion, which is related to the normal development of cells and the stability of the P65 protein, but also participates in the gliding movement of *M. pneumoniae*; as a result, *M. pneumoniae* can move from the tips of epithelial cilia in the bronchioles of the upper respiratory tract to the surface of the host cells. Overall, in the pathogenic process, the P30 protein is located at the tip of *Mycoplasma pneumoniae*’s terminal adhesin and plays a crucial role in signal transduction to host cells, cell adhesion, and motility. The P30 protein shares some sequence homology with the P1 protein and is also a major adhesin factor for *Mycoplasma pneumoniae*. The P30 protein participates in the adhesion of *Mycoplasma pneumoniae* to sialoglycoproteins and sulfated glycolipids on the surface of host cells, triggering changes in host cell metabolism and ultrastructure. In addition, the P30 protein is highly immunogenic, and antibodies can be detected in the serum of infected individuals, providing new ideas for serologic diagnosis of *M. pneumoniae* infection and vaccine development. Despite significant progress in the study of P30 protein, several important unresolved issues remain: The exact three-dimensional structure of P30 protein and its relationship with function are still unclear; the molecular mechanism by which P30 protein participates in cell adhesion is not fully understood; the regulatory mechanisms of immune responses induced by P30 protein require further investigation; and the specific role of P30 protein in the pathogenic process of *Mycoplasma pneumoniae* needs to be elucidated. To address these gaps in P30 protein research, we can utilize protein structural biology methods such as X-ray crystallography and electron microscopy techniques to determine the high-resolution three-dimensional structure of P30 protein. We should also conduct studies on the interaction between P30 protein and host cell receptors to further clarify its cell adhesion mechanism. Through immunological experiments and animal model studies, we can delve into the regulatory processes of immune responses induced by P30 protein. Additionally, by integrating genetics, genomics, and proteomics approaches, we can comprehensively analyze the role of P30 protein in the pathogenic process of *Mycoplasma pneumoniae*. The following article provides a detailed description of the P30 protein in terms of its structure, mutational typing, function and use in serologic diagnosis and vaccine preparation.

## 2 Structure and typing of the P30 protein

### 2.1 Structure of the P30 protein

#### 2.1.1 Gene structure of the P30 protein

The whole genome of *M. pneumoniae* has been sequenced, and this genome is 816,394 bp in size and encodes approximately 700 open reading frames (ORFs). The genome of *Mycoplasma pneumoniae* exhibits a high abundance of A + T, with merely 39–40% being G + C. Additionally, it lacks the genes essential for TCA cycle recycling, cell wall formation, amino acid biosynthesis (*Mycoplasma pneumoniae* fundamentally relies on an obligate parasitic lifestyle and requires exogenous essential metabolites), as well as nucleotide biosynthesis(Meseguer et al., 2003; Himmelreich et al., 1996; Razin et al., 1998). *M. pneumoniae* uses biased codons, and its wild-type genes are not suitable for expression in *E. coli*. Among them, the UGA codon is a termination codon in normal organisms, while in *M. pneumoniae*, the UGA codon encodes tryptophan (Kannan and Baseman, 2000; Citti et al., 2020; Halbedel and Stulke, 2007); this phenomenon has been resolved by multiple mutation reactions (Hames et al., 2005). Although the P30 protein only has a UGA codon at position 16 of its amino terminus, the yield of clonally expressed P30 proteins is low, which may be related to the presence of more rare codons (e.g., AGA and AGG for arginine) in the genome; this is among the biggest reasons why P30 proteins are not well studied (Varshney et al., 2008). Currently, some scholars have designed and obtained the optimized P30 protein-encoding gene sequence by codon optimization to achieve efficient expression in *E. coli*, but the feasibility of this method needs to be further explored.

**Figure 1:**
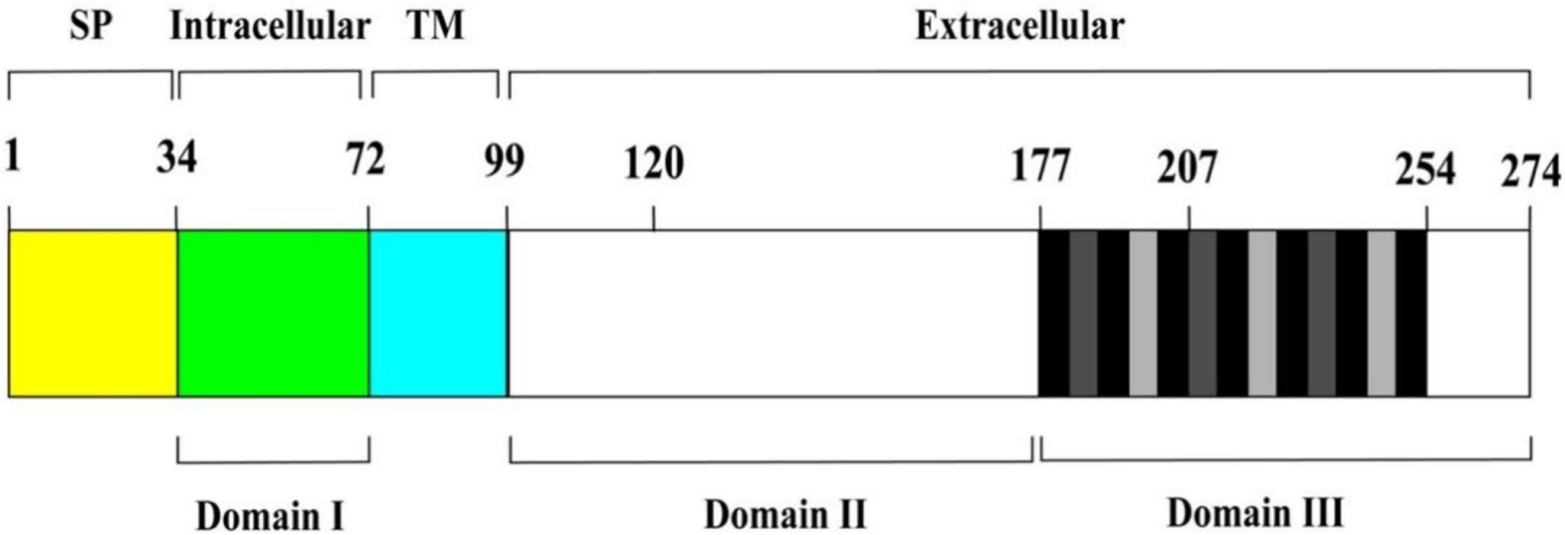
Schematic diagram of P30 protein structure. From left to right, the brackets above indicate the predicted signal peptide (SP) (yellow area), intracellular (green area), transmembrane (TM) (cyan area), and extracellular regions (remaining area) of P30. Domains I, II, and III are represented by the black horizontal lines below. Vertical black and grey bars within domain III correspond to P-rich repeat motifs PGMAPR (black), PGMPPH (light grey) and PGEPPQ (dark grey).

Adhesion protein P30 (MPN453) has a genome size of 825 bp, is located within the HMW genome, encodes 275 amino acids, and has a molecular mass of approximately 29.7 kDa and has a G + C content of 54.4%, which is similar to the 53.5% G + C content of the P1 adhesion protein (Dallo et al., 1990). P30 protein expression requires a promoter-like region upstream of P21 in the HMW genome, and a regulatory sequence 13 bp downstream of the P30 gene is also associated with HMW3 expression, i.e., P30 and HMW3 transcription are interdependent. The C-terminus of the P30 gene has three different proline-rich repeats, each consisting of six amino acids, with repeat A region PGMAPR (occurring seven times), repeat B region PGMPPH (occurring three times), and repeat C region PGEPPQ (occurring three times) (as shown in Figure 1; Chang et al., 2011a). Proline residues are highly conserved in the peptide chain and help regulate transmembrane proteins, playing an important role in protein structure and function. In addition, the amino acid sequence homology between this repeat sequence region and the carboxyl terminus of the P1 protein is between 55 and 67% (Dallo et al., 1990; Baseman, 1993). The carboxyl terminus also possesses amino acid sequence homology with eukaryotic proteins, such as fibrinogen, collagen, keratin and vitronectin, which are highly pathogenic and immunogenic. Gene structure determines protein function, and this high degree of sequence identity between the P30 and P1 proteins further validates the topological and functional similarity of the two proteins (Dallo et al., 1990). Thus, the P30 protein, like the P1 protein, includes three structural domains.

#### 2.1.2 P30 protein secondary and tertiary structure prediction

**Figure 2:**
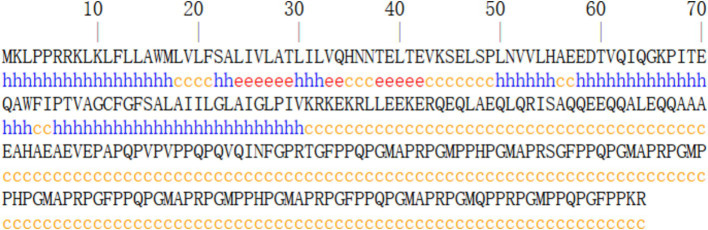
Prediction of secondary structure of P30 protein. Predictions suggest that the secondary structure of the P30 protein is dominated by alpha helices and random coiling.

**Figure 3:**
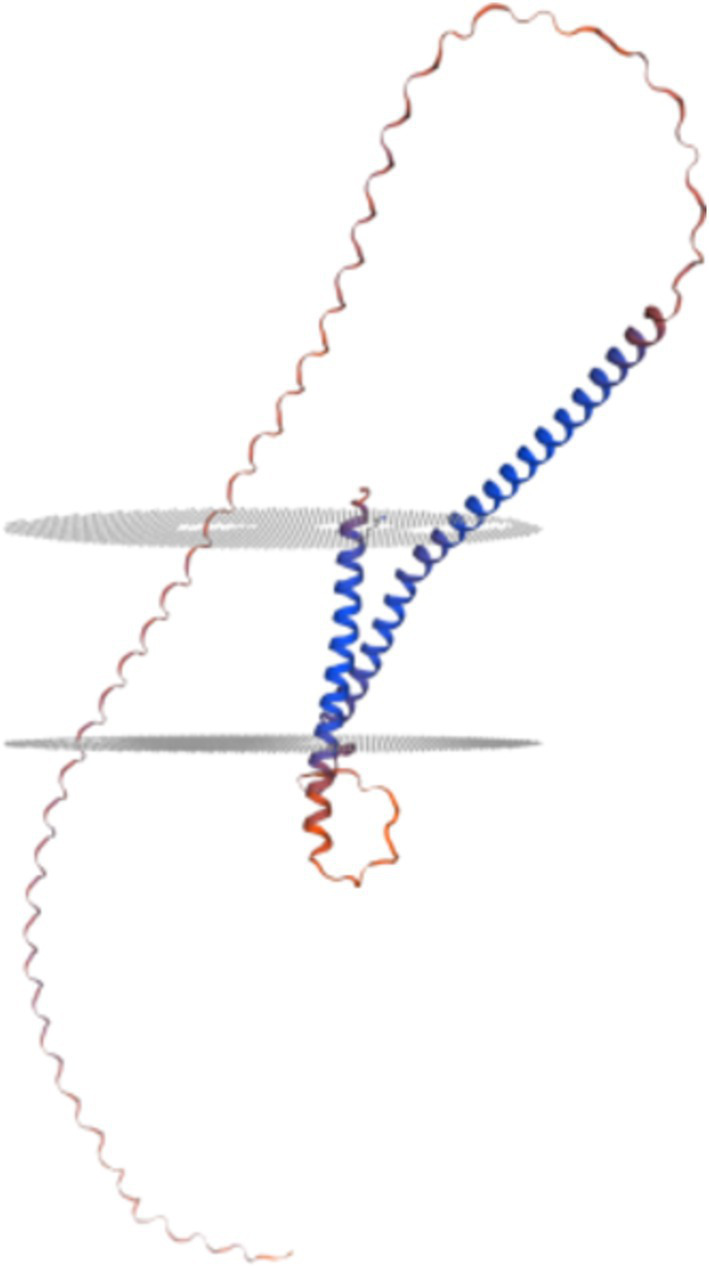
Prediction of tertiary structure of P30 protein. This suggests that the P30 protein is a twice-transmembrane protein.

Analysis using SOPMA^1^ software revealed that the P30 protein’s secondary structure comprises 25.18% *α*-helices, 4.74% extended chains, and 70.07% random coils, indicating a dominance of α-helical and random coiled regions (Figure 2). Tertiary structure prediction via SWISS-MODEL^2^ indicated that P30 is a transmembrane protein with two membrane-spanning regions (Figure 3).

### 2.2 Structural domains of the P30 protein

The protein is a transmembrane protein anchored to the cytosolic membrane of *M. pneumoniae* by its carboxyl terminus and consists of the following parts: the intracytoplasmic region of *M. pneumoniae* (structural domain I), the transmembrane region (TM), and the extracellular domains (structural domain II and structural domain III). The different structural domains correspond to the different functions of the P30 protein (Figure 1). The C-terminus is located in structural domain III, and the N-terminus may be located in structural domain I. P30 structural domain I consists of a putative signaling sequence and an intracytoplasmic region that may be the surface-bound adhesive component connecting the core of the terminal organelle to the C-terminal structural domain of the P30 protein. Alterations in structural domain I may cause conformational changes in its extracellular structural domain, and deletion of structural domain I results in loss of gliding motility and cell adhesion. The transmembrane region TM shows a high degree of conservation with structural domain II, and the carboxy-terminal region of structural domain III contains 13 proline-rich repeats, both of which are similar to the homologs of the P30 protein in other *Mycoplasma gallisepticum* MGC2 protein and *Mycoplasma genitalium* P32 protein (Chang et al., 2011a; Hnatow et al., 1998; Reddy et al., 1995). The P30 TM region separates structural domains I and II, and the TM structural domain includes a GxxxG sequence (G92 to G96), which is associated with interactions between the TM helices and contributes to the distribution of the P30 protein across the *M. pneumoniae* membrane (Russ and Engelman, 2000; Curran and Engelman, 2003). Substitutions within structural domain II or deletions within structural domain III reduce the motility and cell adhesion of *M. pneumoniae*. The proline-rich repeats in structural domain III are needed for the stabilization of P65 (Hasselbring et al., 2005), and deleting this repetitive sequence region, which is extremely antigenic, may have a significant impact on the structural and functional properties of the P30 protein (Seto et al., 2001). Changes in the structural domains are closely related to functional stabilization, and deleting and mutating gene sequences within the structural domains result in different mutant isoforms of the P30 protein (Table 1).

**Table 1:** Mutant isoforms of P30 protein.

P30 derivative(s)		Phenotype					Reference
		P30 molecular weight	P30 stability	HMW-1 stability	HA	Gliding	
Complete gene structure	WT	30 kd	+	+	+	+	Dallo et al. (1990) and Baseman (1993)
	II-3	30 kd	–	+	–	–	Chaudhry (2007), Chang et al. (2011a), Hasselbring et al. (2005), Dallo et al. (1996), Williams et al. (2020), and Romero-Arroyo et al. (1999)
	II-15	30 kd	–	+	–	–	Dallo et al. (1996)
Incomplete gene structure	II-7	25 kd	+	+	+	+	Chaudhry (2007), Chang et al. (2011a), Hasselbring et al. (2005), and Romero-Arroyo et al. (1999)
	M6	25 kd	–	–	–	Unknown	Seto et al. (2001), Layh-Schmitt et al. (1995), and Hahn et al. (1998)
	M7	22 kd	–	+	–	Unknown	Seto et al. (2001) and Layh-Schmitt et al. (1997)

### 2.3 Mutant isoforms of the P30 protein

Characterization of blood adsorption-negative (HA^−^) mutants identifies several terminal organelle proteins, including P30, the deletion of which results in developmental defects in *M. pneumoniae* and reduced adhesion to host cells, as well as loss of gliding motility (Krause et al., 1982). In previous studies, protein profiles were analyzed by one- and two-dimensional acrylamide gel electrophoresis, and *M. pneumoniae* noncellular adhesion mutants were classified into four categories. Among these categories, the P30 protein was mainly absent in the class II noncellular adhesion mutant *M. pneumoniae*, which had a shifted-code mutation in its gene (Baseman, 1993; Romero-Arroyo et al., 1999). In the first subclass of P30-deficient mutants, the P30 structural gene is intact, and the sequence of the P30 gene is unaltered relative to the wild-type P30 gene, but no P30-associated peptide can be synthesized (e.g., mutant II-3 or II-15). A second subclass of mutants has a partial deletion of the P30 gene, resulting in the expression of a 25 kDa peptide (681 nucleotides encoding 227 amino acids with an estimated molecular mass of 24,823 Da), and this P25-truncated peptide lacks eight of the 13 proline-rich amino acid repeats at the carboxy-terminal end (e.g., mutant II-7). Dallo S F et al. found that antibodies to human fibrinogen produced by immunized goats cross-reacted with wild-type P30 protein and the P25 truncated peptide of mutant II-7 but not with mutant II-3 (Baseman, 1993; Dallo et al., 1996). In addition, Layh-Schmitt G et al. identified a P30-truncated peptide protein that lacked the HMW-1 protein and exhibited spontaneous blood adsorption properties (the M6 mutant), and the P30 gene was only approximately 200 bp short. This mainly occurred because the missing HMW-1 protein is extremely useful for cell adhesion, as mutants lacking all five cytoskeletal proteins (HMW1-5) are cell adhesion-negative and nontoxic (Seto et al., 2001; Layh-Schmitt et al., 1995; Hahn et al., 1998). The M7 mutant without adhesion properties expresses a 22 kDa truncated product of the P30 gene (Layh-Schmitt et al., 1997), the truncated 22 kDa protein can be recognized by antibodies against the P30 protein, its C-terminal deletion of 12 proline-rich repeats, and *M. pneumoniae* mostly exhibits a branched morphology (Seto et al., 2001). Different kinds of mutant isoforms result in deletion of one or more functions of the P30 protein, and probing the complete gene structure of the P30 protein is imperative for clarifying its functional characteristics.

## 3 Functional exploration of the P30 protein

**Figure 4:**
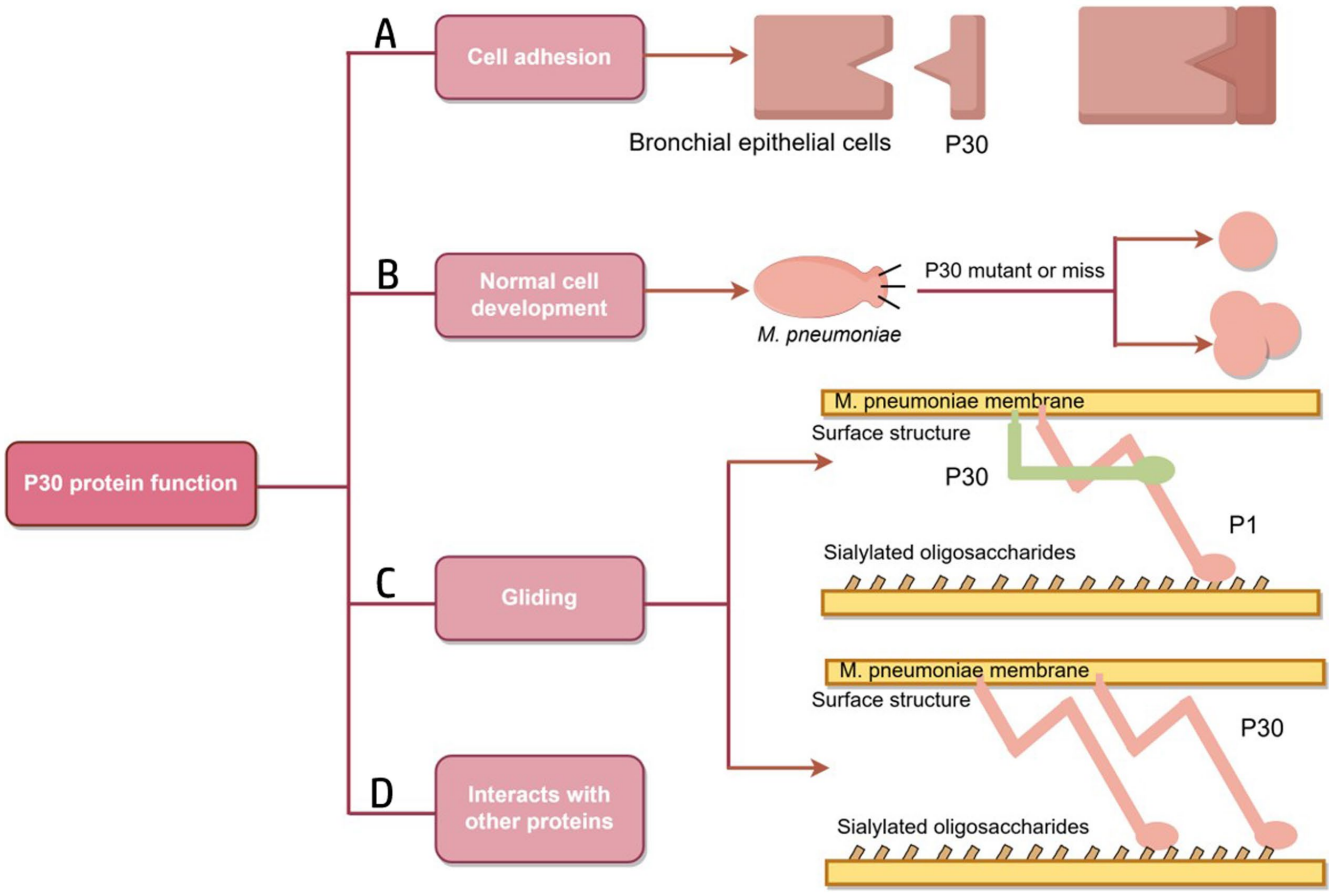
Schematic diagram of P30 protein function. **(A)** A folded conformation is formed; the P1, B and C proteins form an adhesion complex, recognize receptors on host cells and bind to host cells. **(B)** Mutation or deletion of P30 protein results in the transformation of *M. pneumoniae* into oval or multidivision cells. **(C)** The role of P30 protein in the gliding mechanism may be similar to that of Gli349, an adhesion protein of *M. mobilis*, which has a tip structure similar to “foot,” binds to host cell surface receptors and directly drives the mycoplasma to slide on the surface.Similarly, the P30 protein may play a role in Gli521, driving the P1 adhesin that may have a Gli349 function. **(D)** There are interactions between P30, the P1, P65 and HMW3 proteins (Figure by figdraw).

The P30 protein associated with the *M. pneumoniae* attachment organelle is associated with adhesion and motility, and mutations negatively affect cell morphology, adhesion, gliding, and virulence (Relich and Balish, 2011; Figure 4). Mutants lacking the P30 protein are defective in adhesion and motility and are avirulent.

### 3.1 P30 proteins and cell adhesion

**Figure 5:**
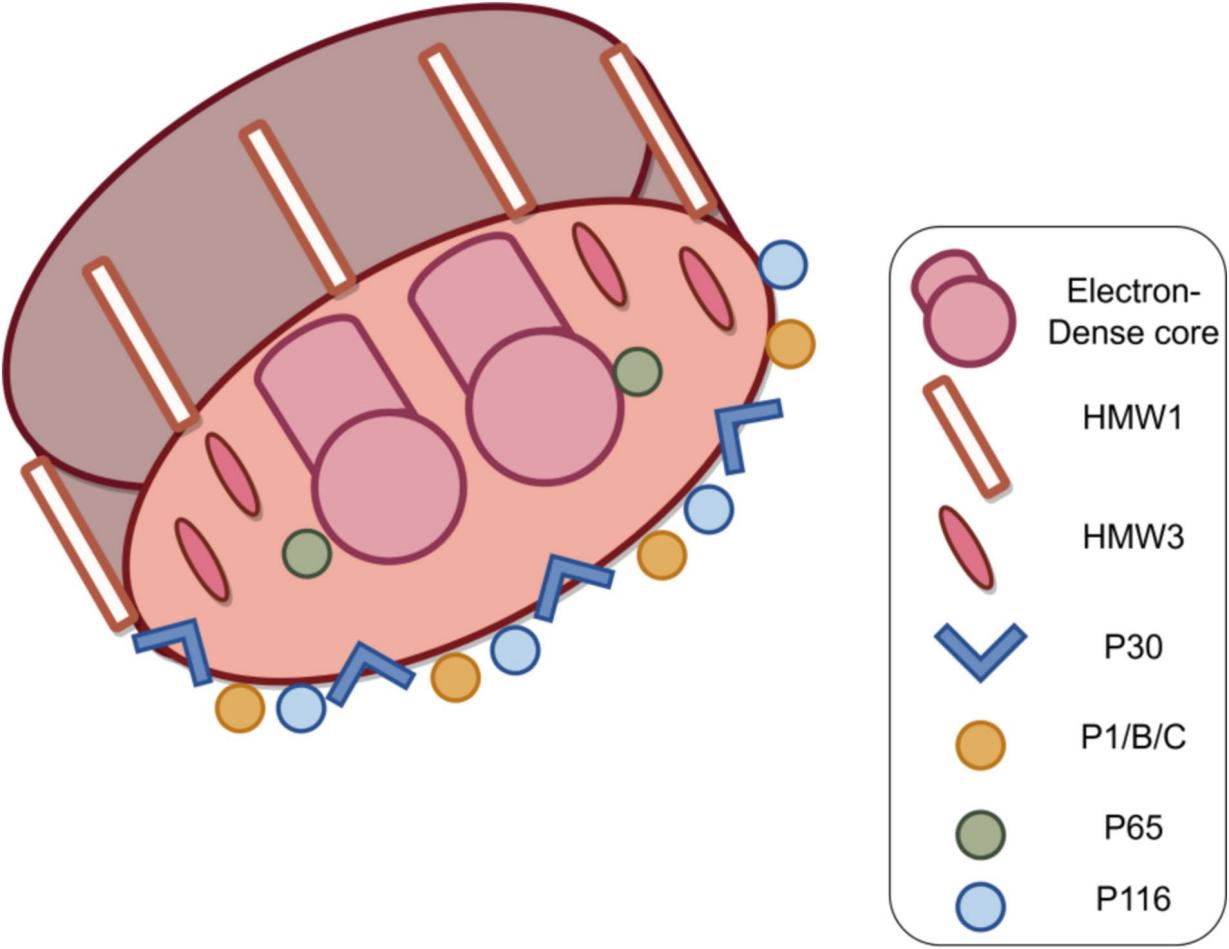
Structure of the *M. pneumoniae* major adhesion proteins and accessory proteins. *M. pneumoniae* adhesion proteins include major adhesion proteins the P1, P30, P116 and P65 proteins, as well as auxiliary proteins P40 and P90 (protein B and C) and high molecular weight protein HMW1-3. Together, these components form a characteristic high electron-density “adhesive protein complex” (Figure by figdraw).

In *M. pneumoniae* infections, adhesion is the major virulence factor in pathogenesis, and adhesion-deficient mutant strains are usually avirulent (Razin, 1999; Hansen et al., 1981). Mycoplasma cell adhesion to the respiratory epithelium is critical for tissue colonization and subsequent pathogenic mechanisms (Chaudhry, 2007; Krause et al., 1982). Adhesion function is primarily mediated by the tip organelle of *M. pneumoniae*, a polar, differentiated, cytosolic-connected portion of the mycoplasma with a complex electron-dense core (Hegermann et al., 2002; Mayer, 2006; Chang et al., 2011b). Cell adhesion is a highly complex multifactorial and collaborative process that requires a set of adhesion proteins in the tip organelles of *M. pneumoniae*, such as P30, the P1, P116, and HMW1-3 (Figure 5). P30 is an intact transmembrane protein located in clusters at the distal end of the tip organelles (Nakane et al., 2015; Krause, 1996). The adhesion function of *M. pneumoniae* requires the P1 protein to be translocated to its terminus, at which it forms an adhesion complex with the P30 and B and C proteins and undergoes a folded conformation for binding to host cell receptors (Suzuki et al., 2017; Layh-Schmitt and Herrmann, 1994). The P30 and P1 proteins can bind directly to salivary acidified oligosaccharide and sulfated oligosaccharide receptors, both of which are essential for *M. pneumoniae* (Williams et al., 2020; Nakane et al., 2015; Krause et al., 2018). *M. pneumoniae* adheres to abiotic surfaces and/or biofilms in association with the *M. pneumoniae* major apical organelle proteins P1 and P30 (Curran and Engelman, 2003). It has been demonstrated that in mutant II-3 with P30 protein deletion, the P1 protein is present at wild-type levels on the surface of the mycoplasma but forms a functional adhesion complex that is defunct (Yu and Fei, 2016; Seto and Miyata, 2003). Deleting the structural domain I of the P30 protein in the cytoplasm has little effect on the stability and localization of the protein but leads to loss of cell adhesion function (Chang et al., 2011a). Substitutions or deletions within structural domains II or III reduce the ability of the P30 protein to adhere to host cells, and antibodies against structural domain III inhibit cell adhesion (Hasselbring et al., 2005; Schurwanz et al., 2009). The proline-rich repeat sequence region is critical for adhesion-related functions. The absence of this region, which is highly antigenic, may help *M. pneumoniae* evade the immune system because reduced adhesion facilitates the spread of *M. pneumoniae* from one host cell to another (Layh-Schmitt et al., 1997).

### 3.2 The P30 protein is associated with normal cell development

Because mycoplasma cells lack a cell wall and possesses a thin layer of a cell membrane package, their shape is mostly spherical, and occasionally also pear-shaped, bottle-necked, filamentous or spiral filamentous and so on (Razin et al., 1998), microcolonies are also a form of pathogenic mycoplasma (Rakovskaya et al., 2019). When grown on inert surfaces, wild-type *M. pneumoniae* are spindle-shaped, usually 1.0–2.0 mm in total length and approximately 0.3 mm in diameter. The bacteria possess a long, conical cellular extension of 300 nm (the tip organelle) at one end and a longer, slender filamentous extension at the other end, as shown in Figure 5. *M. pneumoniae* is predominantly characterized by binary fission propagation, and its adhesion organelles may play a role in cell development. Romero-Arroyo C E et al. demonstrated that the P30 protein is essential in the morphogenesis of *M. pneumoniae*. Wild-type *M. pneumoniae* usually forms a conical tip structure and a meadow-like tail structure within the first hour of development. Mutant II-7, which produces a truncated P30 protein, exhibits an abnormal morphology in the early stages of culture but then becomes indistinguishable from the wild type. On the other hand, mutant II-3, which completely lacks the P30 protein, exhibits a highly variable morphology and forms ovoid or multidivided cells with loss of the conical tip structure. The C-terminal proline-rich repeat structure of the P30 protein is not associated with functions related to cellular development. The N-terminal structural domain of P30, along with other cytoskeletal elements, plays a role in the development of the tip organelles or in the process of chromosome division (Romero-Arroyo et al., 1999).

### 3.3 P30 protein and gliding

In the *M. pneumoniae* phylogenetic group, different species glide at different speeds, implying that some component of the locomotor apparatus regulates velocity (Hatchel and Balish, 2008). The phenotype of the P30 protein-deficient *M. pneumoniae* mutant exibits a branched morphology instead of the filamentous spindle morphology of wild-type *M. pneumoniae*. Based on the current study, the filamentous extension morphology of wild-type *M. pneumoniae* cells may contribute to the function of cell gliding, which binds to salivating oligosaccharides on solid surfaces and glides in the direction of membrane protrusion (Hasselbring et al., 2005; Kasai et al., 2013; Williams et al., 2018). The cellular extension, the tip organelle of *M. pneumoniae* (an enormous, complex, conformationally flexible molecular machine), may contain a dynamic motor that is much larger than flagellum contained most motile bacteria; in addition, this motor may be an important reason why *M. pneumoniae* can glide, as suggested by Seybert et al. (2018), Henderson and Jensen (2006), and Hasselbring and Krause (2007). The adhesion proteins P1 and P30 are involved in the gliding motility of *M. pneumoniae* through conformational changes and their displacements, and the P30 protein is localized at the end of the tip organelle (Seto et al., 2005). Morrison-Plummer et al. (1986) assessed mycoplasma protein function by homologous gene transformation methods to propose the hypothesis that P30 proteins may play a role in gliding mechanisms similar to that of *Mycoplasma mobile* and adhesion protein Gli349, as both bacteria perform similar adhesion functions (Relich and Balish, 2011). In *M. mobile*, the Gli521 protein transmits propulsive force to the Gli349 protein, which possesses a “foot”-like tip structure that binds to a host cell surface receptor (Miyata, 2010; Toyonaga et al., 2021). Another possible function of the P30 protein is that it interacts with proteins that directly drive movement and promote their activity (Relich and Balish, 2011). Similarly, P30 may play this role of Gli521 in *M. pneumoniae*, whereas some other attachment organelle proteins, such as major adhesin P1, play the role of Gli349 (Relich and Balish, 2011). Alterations in the locomotor performance of *M. pneumoniae* may also directly affect biofilm formation, leading to nonnormal morphogenesi*s* (Feng et al., 2018).

### 3.4 Association of the P30 protein with other proteins

The P65 protein, which is a component of the cytoskeleton that is insoluble in Triton X-100, lacks an obvious signal sequence and, together with P30, is localized to the distal end of the tip organelles on the membrane surface of wild-type *M. pneumoniae* (Seto et al., 2001; Hasselbring et al., 2006; Jordan et al., 2001; Kenri et al., 2004). The genes that encode P65 and P30 are located on the CRL and HMW manipulators, respectively (Jordan et al., 2001; Kenri et al., 2004; Proft et al., 1995). Low levels of P65 protein in *M. pneumoniae* have been associated with deletion and truncation of P30 (Jordan et al., 2001); in contrast, truncation of P65 protein affects the cell adhesion, gliding motility, and surface dynamics functions of P30 protein. P1 proteins require P30 proteins to function, and full-length P65 proteins can contribute to a stable connection between P1 and P30 proteins (Hasselbring et al., 2012). The gene that encodes the P30 protein is located upstream of the HMW3 gene, and the two are co-transcribed. The P30 protein cannot localize to terminal organelles in the absence of HMW3, but its levels are stabilized (Krause and Balish, 2004; Willby and Krause, 2002; Table 2).

**Table 2:** Association of P30 protein function with domains and other proteins.

P30 protein function	Structural domain	Same acting protein	Reference
Cell adhesion	III(proline-rich repeat domain)	P1, P116, P65, B, C, HMW1-3	Razin (1999), Hansen et al. (1981), Hegermann et al. (2002), Mayer (2006), Chang et al. (2011b), Nakane et al. (2015), Krause (1996), Layh-Schmitt and Herrmann (1994), Krause et al. (2018), and Schurwanz et al. (2009)
Nomal cell development	I	P65, FtsZ	Razin et al. (1998) and Krause and Balish (2004)
Gliding	III	P1, EF-Tu, FtsZ, DnaK	Hatchel and Balish (2008), Kasai et al. (2013), Williams et al. (2018), Seybert et al. (2018), Henderson and Jensen (2006), Hasselbring and Krause (2007), Seto et al. (2005), Morrison-Plummer et al. (1986), Miyata (2010), Toyonaga et al. (2021), and Feng et al. (2018)
Interacts with other proteins	–	P1, P65, HMW3	Hasselbring et al. (2006), Jordan et al. (2001), Kenri et al. (2004), Proft et al. (1995), Hasselbring et al. (2012), and Willby and Krause (2002)

## 4 Serological diagnostic role of the P30 protein

Similar to the P1 protein, the P30 protein is a transmembrane protein that binds to host cell receptors and is an important immunogenicity factor (Nakane et al., 2015; Leith et al., 1983). Schurwanz et al. (2009) experimentally demonstrated that the P30 protein (amino acids 17 ~ 274) exhibited significant immunoreactivity against *M. pneumoniae*-immunized guinea pig sera and *M. pneumoniae*-positive patient sera. The results obtained by Varshney et al. (2008) suggests that the P30 protein can be used as an antigen along with other adhesion proteins for immunoserological diagnosis of *M. pneumoniae* infection. The C-terminus of P30 proteins is exposed on the surface of *M. pneumoniae*, has a proline-rich repeat sequence, and exhibits a high potential to generate an immune response in sera from patients infected with *M. pneumoniae*. The recombinant maltose-binding protein (MBP)-P30B fusion protein could be recognized by *M. pneumoniae*-infected patient sera, and the protein could produce strong immunogenicity in mice. Moreover, the purified MBP-P30B fusion protein ELISA exhibits high specificity and sensitivity. The P30 protein can be used together with two other adhesion protein molecules, P1 and P116, for the development of sensitive assays for the diagnosis of infection with *M. pneumoniae* (Tabassum et al., 2010).

## 5 Role of the P30 protein in candidate vaccine research

Drug-resistant *M. pneumoniae* is becoming more common in clinical practice, making it increasingly difficult to treat *M. pneumoniae* infections in children. Therefore, effective vaccines to prevent *M. pneumoniae* infections are urgently needed for clinical environments. Mnay types of *M. pneumoniae* vaccines are available, which mainly include inactivated vaccines, live attenuated vaccines, and protein subunit vaccines (involving the P1 protein, the P30 protein, the P116 protein, and the CARDS toxin), and recombinant DNA vaccines (Jiang et al., 2021). Adhesion protein-mediated binding of mycoplasma to host cells is a critical step in colonization and subsequent pathogenesis, and directly blocking *M. pneumoniae* colonization is the most desirable vaccine mechanism (Schurwanz et al., 2009). In addition, *M. pneumoniae* infection of humans elicits a strong immune-antibody response that is directed against many adhesion proteins in the tip structures on the surface of *M. pneumoniae* (Dumke and Jacobs, 2016). Therefore, the design and production of vaccines targeting adhesion proteins is the optimal choice to inhibit the colonization of respiratory mucosa by *M. pneumoniae*. The immunodominant fragments of vaccine candidate proteins were screened by secondary structure and structural domain prediction, antigenic epitope prediction, and special codons, and the P30 protein was designed as a vaccine candidate based on these principles.

**Figure 6:**
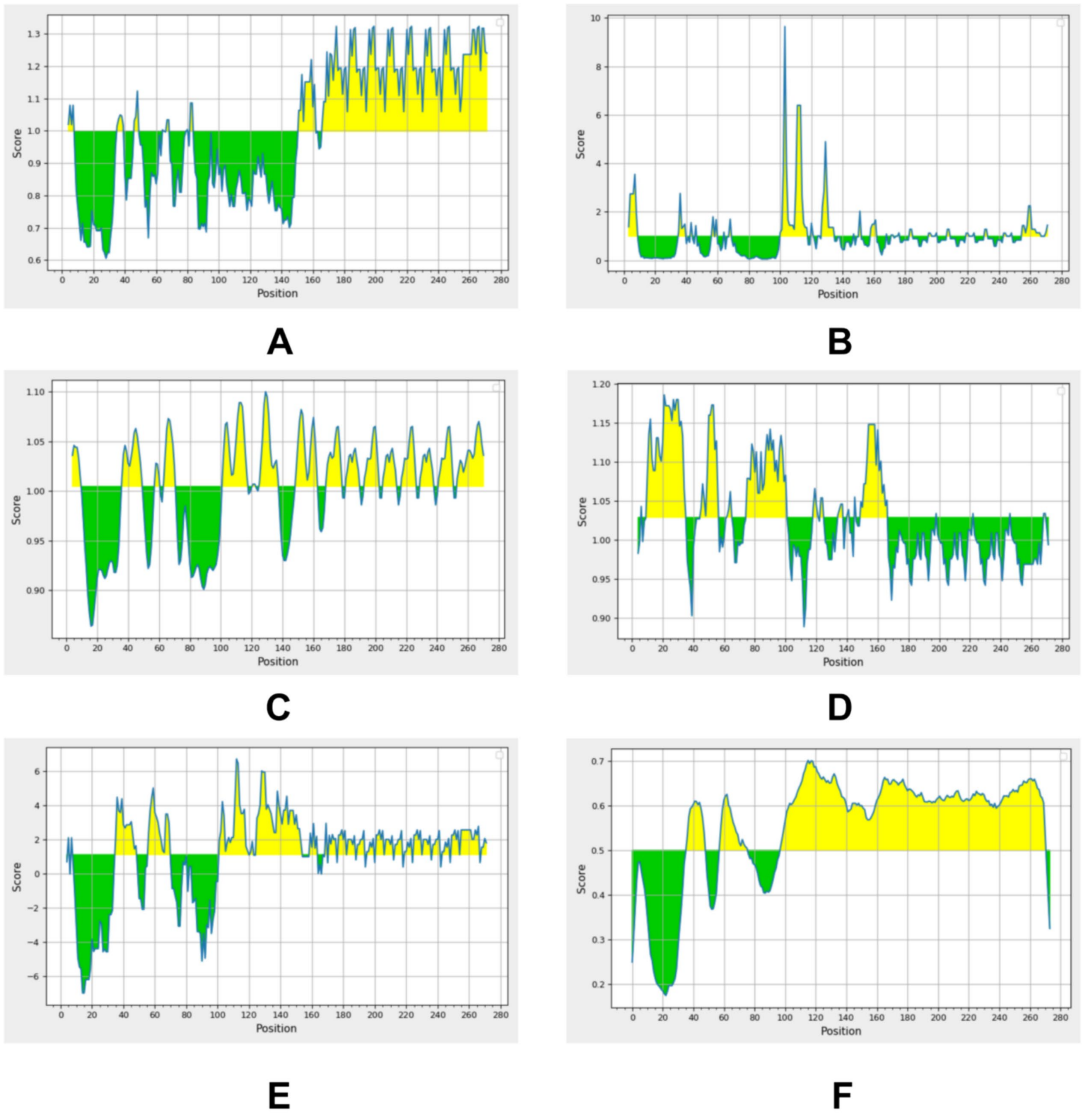
Residues with scores above the threshold (indicated in yellow in the figure) are predicted to be part of the epitope. **(A)** Beta-turn prediction result; **(B)** Surface accessibility prediction result; **(C)** Flexibility prediction result; **(D)** Antigenicity prediction result; **(E)** Hydrophilicity prediction result; **(F)** Bepipred linear epitope prediction 2.0 result.

Given that *M. pneumoniae* primarily induces a humoral immune response, B-cell antigen epitope prediction was crucial. Utilizing the Immune Epitope Database (IEDB) tools,^3^ we analyzed the P30 protein for *β*-turns, surface accessibility, flexibility, antigenicity, hydrophilicity, and bepipred linear epitope. Regions exceeding the baseline in the analysis graphs (highlighted in yellow) indicated a higher likelihood of forming epitopes (Figure 6). Additionally, we employed ABCpred software^4^ with a default threshold set at 0.5; prediction results scoring above this threshold were considered significant. By jointly analyzing the B-cell antigenic epitopes predicted by both software programs, we identified seven high-scoring dominant B-cell epitopes (Table 3).

**Table 3:** Prediction of B cell epitopes of P30.

Serial number	Starting amino acid site	Ending amino acid site	Sequence	Sequence length (bp)
1	148	156	VEPAPQPVP	9
2	156	162	PVPPQPQ	7
3	158	164	PPQPQVQ	7
4	195	201	PGFPPQP	7
5	219	225	PGFPPQP	7
6	243	249	PGFPPQP	7
7	265	272	PPQPGFPP	8

*Mycoplasma pneumoniae* mutants that lack the encoded adhesion protein P30 gene are nonadhesive and pathogenic, suggesting that the P30 protein may be an ideal candidate target for clinical vaccines (Blötz et al., 2020). Szczepanek et al. (2012) constructed a nontoxic mutant P30 adhesion protein as a candidate live attenuated vaccine and evaluated the efficacy of the vaccine by immunizing mice. However, the results were unsatisfactory, and when *M. pneumoniae* live attenuated vaccine-immunized mice were challenged with the virulent strain, the disease was exacerbated and complications occurred; these effects were mainly caused by eosinophilic infiltration and helper T-type 17 (Th17) cell-mediated autoimmune or antimicrobial immune responses (Rathore and Wang, 2016; Yang et al., 2014). As a result, research on live attenuated vaccines involves certain shortcomings, and more caution and care are needed in vaccine design. Schurwanz et al. (2009) optimized the immunogenicity and adhesion properties of the antigen by combining partial fragments of the P1 and P30 proteins in a new fusion protein, and antibodies against this fusion protein show promise for effectively inhibiting *M. pneumoniae* adhesion and colonizing host respiratory epithelial cells. Hausner et al. (2013) also optimized immunization by combining the P1-P30 fusion protein with the mucosal adjuvant chitosan, which can reduce the colonization of *M. pneumoniae* in the respiratory tracts of infected animals, and further studies are needed to optimize the fusion protein. Chen et al. (2016) created a fusion recombinant protein P116N-P1C-P30 (abbreviated as MP559) from fragments of three adhesion proteins, the P1, P30 and P116, and MP559-immunized rabbits could produce antibodies against each of the three proteins separately. MP559 reacted with the sera of rabbits immunized with the P1, P30, and P116 separately, and this fusion protein is expected to be a more optimized vaccine candidate.

## 6 Summary and future prospects

Skin and mucous membranes and their secretions are the body’s first line of defense against pathogens (Günther and Seyfert, 2018), and adsorption of *M. pneumoniae* to respiratory epithelial cells through its tip structure is a prerequisite for *M. pneumoniae* pathogenesis. *M. pneumoniae* is an important pathogen that causes respiratory tract infections, and its pathogenic process consists of adherence, proliferation, and release of virulence factors; among them, the adherence process is an important link of *M. pneumoniae* infection (Jiang et al., 2021; Chaudhry, 2007). A series of studies on P30, the main adhesion protein of *M. pneumoniae*, have led to knowledge on the pathogenic mechanism of *M. pneumoniae*, which can be used for the diagnosis, treatment, and prevention of *M. pneumoniae*. The P30 protein is not only involved in the adhesion process of pathogens but also plays an important role in *M. pneumoniae* gliding motility and normal cell development. This paper discusses the progress of research on the structure, function, diagnosis and prevention of the P30 protein of *M. pneumoniae*. Although the genetic structure and amino acid composition of the P30 protein are known, the complete structure of the P30 protein, its interaction with adhesion proteins, its interaction with host cell surface receptors, the relationship between its mutation and *M. pneumoniae* resistance and infection, and the application of the P30 protein in vaccines have not been explored. Similar homologs have not been found for the P30 protein in species other than Mycoplasma, and research to clarify the complete structure of the P30 protein could provide valuable clues on its role in adhesion, gliding functions and virulence. For this reason, we believe that the following methods can enhance our understanding of P30 protein and provide better insights for the diagnosis and treatment of *Mycoplasma pneumoniae*. The purification of P30 protein was carried out using an improved Virus Overlay Protein Binding Assay (VOPBA) and Liquid Chromatography-Mass Spectrometry (LC–MS) to screen for specific interaction proteins binding to the membrane of BEAS-2B bronchial epithelial cells. The interactions were further verified by adhesion and adhesion inhibition assays, which assessed whether the interaction proteins and their antibodies could inhibit the binding of purified P30 protein and *Mycoplasma pneumoniae* to BEAS-2B cells. Additionally, the levels of adhesion between BEAS-2B cells with knocked down or overexpressed interaction proteins and purified P30 protein or *Mycoplasma pneumoniae* were compared to wild-type cells. We also can utilize SERS technology to further investigate the distinguishing characteristics between different genotypes or phenotypes of *Mycoplasma pneumoniae* strains, laying the foundation for rapid and accurate clinical testing.

## Footnotes

^1^ https://npsa.lyon.inserm.fr/cgi-bin/npsa_automat.pl?page=/NPSA/npsa_sopma.html

^2^ https://swissmodel.expasy.org/interactive

^3^ http://tools.iedb.org/bcell/

^4^ https://webs.iiitd.edu.in/raghava/abcpred/
